# Ascites—IX

**Published:** 1909-08-07

**Authors:** 


### Medicine.

### ASCITES—IX.

### THE DIFFERENTIAL DIAGNOSIS OF THE CAUSE OF ASCITES (*concluded*).

*Ascites due to heart failure from chronic lung disease or from chronic valvular or muscular heart disease* is usually preceded by swelling and œdema of the feet and legs. A careful examination of the heart and lungs, and the detection of signs of valvular disease of the heart, or of chronic bronchitis and emphysema or of fibroid lung would account in a satisfactory manner for the presence of ascites. The clinical picture of chronic heart failure is ultimately so similar, no matter whether the primary cause is renal, pulmonary, or cardiac, that it is often extremely difficult to allocate the failure to its right cause. Two points should receive particular attention in all such cases, namely, the condition of the retinæ and the microscopical character of the urinary deposit.

It should be remembered that any condition of chronic failure of cardiac compensation may produce enlargement of the liver from passive congestion—a "nutmegged" liver. The enlargement may be considerable. The edge of the liver is usually sharp, firm, and well defined; it may reach to the level of the umbilicus in the right nipple line, or even extend below it. The hepatic surface is firm rather than hard, and quite smooth. It will be tender or not tender according as the enlargement from heart failure has occurred rapidly and recently or the reverse. In some cases the viscus may be felt pulsating. In all such cases the urine is concentrated, scanty, high coloured, of high specific gravity, and nearly always albuminous, with but few casts.

*Bright's Disease.*—As we have pointed out, Bright's disease may give rise to ascites in at least four entirely different ways, namely: (*a*) as part of general anasarca, (*b*) as the result of acute "simple" peritonitis, (*c*) as the result of chronic "simple" peritonitis, with or without perihepatitis, (*d*) as the result of backward pressure from failing compensation in a dilated hypertrophied heart.

(*a*) In acute Bright's disease ascites may occur as a part of general anasarca. The feet, legs, scrotum, body, loins, and face may all be swollen and œdematous, and there may be signs of fluid in the pleural and pericardial cavities at the same time. The urine is diminished in total quantity, its specific gravity is high, it may contain a large quantity of blood and albumen, and microscopically it abounds in renal epithelial cells, blood corpuscles, and all kinds of tube casts. In chronic tubal nephritis ascites may also be associated with general anasarca. The patient is usually very anæmic, and the urine may be pale, of lower specific gravity than normal, with abundance of albumen, little or no blood, many hyaline and granular tube casts, but not so many renal epithelial cells and epithelial and blood casts as in the acuter phases of the malady.

(*b*) and (*c*) Acute and chronic peritonitis and perihepatitis as causes of ascites, and their relations to Bright's disease, have already been alluded to.

(*d*) Ascites the result of backward pressure following dilatation of the hypertrophied heart that results from chronic renal fibrosis may be extremely difficult to distinguish from ascites caused by primary disease of the heart or by certain chronic pulmonary complaints. The abundant pale urine of low specific gravity with a trace of albumen, characteristic of granular kidney, becomes changed by heart failure into a scanty urine of high specific gravity, and the albumen in it may become as abundant as it is in acute nephritis. One relies less, perhaps, upon the urine here than upon the condition of the radial artery as to its thickness and tortuosity, the presence of retinal hæmorrhages or the white patches of albumin-uric retinitis, a history of the need to rise more than once at night to micturate although the prostate is normal, or a history of such predisposing causes as chronic alcoholism, high living, gout, or plumbism. The family history is often of considerable assistance also, for arteriosclerosis, granular kidney, and cerebral hæmorrhage all have a tendency to run in families.

Ascites, the result most probably of a very mild form of chronic peritonitis, is liable to occur in cases of *splenomedullary leuchæmia*, *lymphatic leuchæmia*, *Hodgkin's disease*, *splenic anæmia*, and to a less extent in *pernicious anæmia*. In the first four of these five conditions the spleen is likely to be enlarged, and in none of the five is a blood examination likely to be omitted. The condition of the blood itself is pathognomonic in the cases of the two forms of leuchæmia, and in pernicious anæmia. Hodgkin's disease and splenic anæmia will still remain as possibilities if the blood counts are negative, the former being indicated with considerable probability if there are big masses of discrete lymphatic glands to be felt in the neck, axillæ, or groins; whilst splenic anæmia must always remain a difficult diagnosis, made only when every other cause of splenic enlargement has been excluded, and even then often turning out to be only an early stage of cirrhosis of the liver, *i.e.* Banti's disease.

Ascites may follow the rupture of an *ovarian cyst*, or it may be associated with papilloma of the ovary, when there are secondary papillomatous growths in the peritoneum. Ovarian cyst and ascites may therefore coincide with one another. A few years ago a familiar figure in the medical wards at Guy's Hospital (Pye Smith, *Path. Soc. Trans.* xliv) was a woman, about forty years of age, who, towards the end, used to come in several times a month to have her abdomen tapped. Between September 20th, 1884, and April 24, 1893, paracentesis abdominis was performed 299 times. Occasionally the patient used to let out the fluid herself by inserting a knitting-needle into one of the healed punctures which had been made by the trochar and cannula. At the post-mortem examination two large cauliflower-like masses of papillomatous growths were found sprouting from each ovary, and there were a number of secondary growths which were confined chiefly to the parietal peritoneum and to the peritoneum covering the liver, stomach, and spleen. The ascitic fluid in this case was usually clear, straw-coloured, of specific gravity varying between 1,015 and 1,025, and microscopically it contained large round epithelioid cells and club-shaped villi covered with cylindrical epithelium. There were no distant metastases, and clearly the growths were not very malignant. It has, indeed, been stated that if the ovarian papillomata are themselves removed in cases of this sort there is no need to touch those that are scattered over the peritoneum, because these will disappear spontaneously after the primary masses are gone, and the ascites will cease to recur. The importance of correct diagnosis and treatment in such a case is obviously very great.

